# Social demographics and clinical characteristics of referred adult mental health patients to an Australian secure extended care unit: A 5-year retrospective study

**DOI:** 10.1177/00207640251343171

**Published:** 2025-06-12

**Authors:** Partha Das, Emma Robertson, Victoria Harpwood, Stephanie Tierney

**Affiliations:** 1 Adult and Old Age Mental Health Division, Austin Hospital, Heidelberg, VIC, Australia; 2 Nuffield Department of Primary Care Health Sciences, University of Oxford, UK

**Keywords:** Rehabilitation, low secure units, severe mental illness

## Abstract

**Introduction:**

Secure Extended Care Units (SECU) offer low-secure, long-term inpatient rehabilitation for patients with severe mental illnesses. Limited research is available about the profile of patients referred to such units.

**Objective:**

This study aimed to explore the sociodemographic and clinical characteristics of patients referred to Austin Health SECU over a 5-year period in Australia.

**Methods:**

A retrospective study design was used to investigate 121 consecutive referrals. The 98 first-time patient referrals were included in the primary analysis. Descriptive statistics were used with non-parametric comparisons (Chi-square and Fisher’s exact test where appropriate).

**Results:**

Most of the total sample were single males of European ancestry, between 25 and 34 years old, with 10 years or less of education and receiving disability benefits. Schizophrenia was the predominant diagnosis, with 50% having a comorbid personality trait/disorder; substance use was high (82.6%). More than three-fourths had a history of trauma. Physical comorbidity was high (80%), with hepatitis C positivity at 20%. Treatments like Clozapine and Electroconvulsive therapy (ECT) were low. The Median Health of Nations Outcome Scale (HoNOS) was 20 (IQR: 14, 23) and the Life Skills Profile (LSP) was 22.5 (IQR: 16.25, 27).

**Conclusions:**

Referrals showed a high level of psychosocial-physical complexity, with a range of patient needs, service goals, and high psychiatric and interpersonal risk before the referral. The study discusses the need for medium and high-secure beds and a new model of care that integrates Community Care Units (CCU). A trauma-informed approach that creates holistic treatment plans and includes patients and families is indicated. The study makes a case for Universal screening of patients for Bloodborne Hepatitis and treatment for this cohort in an inpatient setting.

## Introduction

Secure Extended Care Units (SECU) were created in the post-deinstitutionalization era (1995–1999) to manage patients with complex and severe mental illness (SMI) within mainstream hospitals (Department of Human Services, 2007). In the Australian context, little information about such units and the profile of referred or waitlisted patients is available. The majority of previous studies have involved patients who were admitted to forensic rehabilitation units in the United Kingdom (low, medium, and high secure; Harty et al., 2004; Killaspy, Marston, et al., 2016). Only a few studies included waitlisted patients (Lavelle, 2007) or those in an Australian low-secure forensic unit (Huang et al., 2023).

### SECU model of care

SECUs provide slow-stream, extended inpatient rehabilitation, utilizing recovery-oriented care principles (State of Victoria. Royal Commission into Victoria’s Mental Health System, 2021). Patients are expected to progress from these structurally locked units to less restrictive environments with a graduated return to community-based care (Department of Human Services, 2007).

Historically, managing bed access has been a significant challenge (Department of Human Services, 2007; State of Victoria. Royal Commission into Victoria’s Mental Health System, 2021). Victoria has 127 SECU beds across the state, 101 in Metropolitan Melbourne, and 26 in rural and regional areas (State of Victoria, 2015), serving a population of six and a half million people (Australian Bureau of Statistics, 2021b). Furthermore, concerns have been raised about the adequate staff skill set, design, and architecture of such units and whether they meet the needs of patients and families (State of Victoria. Royal Commission into Victoria’s Mental Health System, 2021).

### Characterization of patients: Current evidence

Studies conducted to understand accepted patients’ clinical needs and demographic status provide some clinical directions to the referrals received by such secure units (Killaspy, Cardoso, et al., 2016; Melzer et al., 2004a).

Most studies (Berry et al., 2003; Harty et al., 2004; Huang et al., 2023; Neillie et al., 2007) in low, medium and high-secure units found that patients had similar socio-demographics but differed in clinical presentation regarding comorbidity and risk history. The patients across these units were usually male, aged between 18 and 69 years, with long-standing psychiatric histories, and predominantly had a lived experience of schizophrenia with comorbid substance use. Killaspy, Marston, et al. (2016) found a median length of contact with services of 12 years and a median of four previous admissions. Two-thirds of patients were involuntary. Twenty percent had previously been admitted to SECUs. The most prevailing risk profile was the risk of self-neglect and self-harm.

Comorbidity and contact with mental health services varied depending on the secure nature of the receiving unit. Melzer et al. (2004a, 2004b) study in medium-secure units found over three-quarters had personality disorder features. About 84% had been in contact with psychiatric services before referral. Similar findings were made by Berry et al. (2003) study of referrals to high-secure hospitals in the United Kingdom, where the majority of the cohort had a personality disorder (59%). Sixty-nine percent had previous contact with mental health services.

In contrast, studies in forensic low-secure units, such as Huang et al. (2023) study from Australia, showed low rates of previous hospitalization and high comorbidity multi-substance use (65% of patients). Approximately half of the patients were on clozapine and half on depot medications (Huang et al., 2023). Huang et al. (2023) and Neillie et al. (2007) found a low proportion of primary or comorbid diagnoses of personality disorders in low-secure units.

### Demand for inpatient rehabilitation beds

Allison et al. (2018) and T. Edwards et al. (2016) noted the reduction in the number of psychiatric beds in Australia and Europe over the last few decades. In contrast, there has been an increase in the number of psychiatric patients in supported accommodations and prisons. Poor access to inpatient rehabilitation has led to inappropriate use of acute inpatient beds and lengthy delays in finding placements (Allison et al., 2018; Sisti et al., 2015). This leads to premature discharge and, consequently, repeated cycles of hospitalization, homelessness, and transfers between prison and hospitals or disinclination to accept transfers or assertively manage such presentations (Allison et al., 2018; Carroll et al., 2021; Sisti et al., 2015).

Therefore, a study was conducted to investigate the demographic and clinical characteristics of patients referred to the Austin Health SECU over a 5-year period in Australia. Developing an in-depth understanding of the referral cohort will help ascertain the patient profile and guide best practices for services, a model of care, and the needs of these patients. It is crucial to examine the needs of the complex SMI patient cohort so that the right bed can be offered at the right time. This is the first study in Australia to investigate this complex cohort of patients in a non-forensic setting.

## Methodology

A retrospective study design was used. The study period was between January 1, 2019 and December 31, 2023. The period selection was based on the availability of a database of SECU referrals. The study was conducted at Austin SECU, a 25-bed inpatient rehabilitation facility within Austin Hospital in northeast Melbourne. It receives referrals from four Area Mental Health Services (AMHS) serving the Northwest, Northern, Inner East, and Northeast metropolitan regions of Melbourne. It also receives referrals from forensic services if patients have linkages to these geographic regions and, occasionally, from other SECUs across the state. Northeast Area Mental Health Service (NEAMHS) is responsible for the clinical and operational governance of Austin SECU. Together, they serve a catchment with a population of more than 1.3 million residents (Australian Bureau of Statistics, 2021b).

Referrals to SECU are received from SECU liaison officers at each of the mental health services, who nominate individuals assessed as having the most in need of the allocated beds. The patient is assessed for suitability for SECU admission based on a multitude of factors, including severe prolonged illness, extensive psychosocial deficits, patient goals, repeated or protracted admissions to acute inpatient units, poor treatment response, concurrent medical and substance use, service goals, risk profile suitable for management in SECU environment, risk mitigation plans. Factors such as primary personality disorder, moderate to severe Acquired Brain Injury (ABI) or intellectual disability, organic brain disorders, primary substance use disorder and a significant risk to staff are considered exclusion factors.

### Operationalizing variables included in the analysis

The diagnoses in patient files were derived from the 10th edition of the International Classification of Disease (ICD-10; World Health Organization, 2004). The years of contact with AMHS were calculated from the initial contact. The total number of admissions and years since the first involuntary admission were manually calculated using the Client Management Interface (CMI). Homelessness was categorized using the Australian Bureau of Statistics (ABS) homelessness operational group criteria 2021 (Australian Bureau of Statistics, 2021a).

Outcome measures of the Health of Nations Outcome Scale (HoNOS; Wing et al., 1996) and Life Skills Profile (LSP) data (Rosen et al., 1989) were used to characterize a typical referred patient to SECU and accepted patient. The HoNOS and LSP were gathered from patient files on admission and CMI.

The study considered 121 consecutive referrals to the SECU during the study period for 98 patients. Some patients had multiple referrals during the study period (2019–2023). The researchers elected to focus on first-time referrals as opposed to repeat referrals. Re-referrals to SECU arose as a result of operational barriers, like lack of beds available at the time of initial referral, patients being incarcerated and refused bail, and community patients being disengaged and unable to be located. In some instances, hospitalized patients were discharged from the hospital by Mental Health Tribunals with community orders as the least restrictive treatment option.

**Figure 1.**
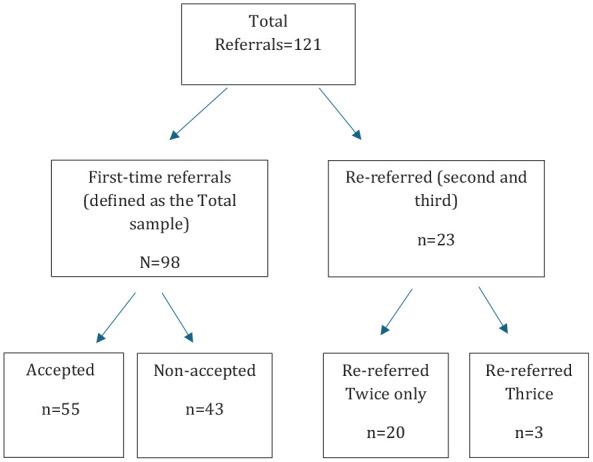
Distribution of patients to the total sample group, re-referral and accepted/non-accepted patient groups.

All the first referrals during the study period were categorized as the Total sample (*N* = 98). The unit of analysis was patients. The total sample was further classified into accepted (*n* = 55) and non-accepted groups (*n* = 43). Data analysis was conducted for the total sample and the subgroups (accepted and non-accepted patients) Figure 1.

### Data collection

The SECU referral database was reviewed and coordinated with the electronic medical records analytics team (EMR) and AMHS-SECU liaison coordinators to ensure completeness. Researchers conducted a further search of the Victorian CMI database, which records patient diagnoses that have been given at discrete points in time (admission, discharge, crisis assessment, and mandated review timeframes). Key variables like substance use, trauma history, forensic involvement, family violence (FV), polypharmacy and Electro Convulsive Therapy (ECT) were chosen based on the clinical characteristics commonly associated with the SECU referral population. These variables reflect the high level of clinical and psychosocial complexity typically observed in this group. The selection was informed by a working hypothesis that patients referred to SECU represent a particularly complex, vulnerable and enduring treatment needs subgroup within the broader mental health service system.

### Statistical analysis

Data analysis was conducted using IBM SPSS for Macintosh Version 28 (IBM Corp, 2021). A complete case analysis was conducted. The Mann–Whitney test was used to compare various continuous variables across the groups of accepted and non-accepted participants in the program, for example, socio-demographic and clinical profiles across two groups. Chi-square tests for independence were used to compare categorical variables like diagnosis with Fisher’s corrections where applicable. Spearman’s correlation coefficient was estimated to measure the strength of the association between socio-demographic and clinical factors with acceptance into SECU. Significance testing was conducted at the 0.05 alpha level, with variables yielding a *p*-value < .05 considered statistically significant.

## Results

### Demographic characteristics

The median age of the referred Total sample was 34 years (IQR: 28.5–39.5). Most referrals came for patients in a younger age group (51%, 18–34 years of age). Most were single (82.7%), males (66.3%), of European ethnicity (39.8%), from English-speaking backgrounds (80.6%). Most were educated to secondary school level, having completed at least 10 years of school education (71.4%). The Accepted and Non-accepted groups were not statistically different for socio-demographic variables (Table 1).

**Table 1.** Socio-demographic for the total sample, accepted and non-accepted groups.

Socio-demographics	Total sample (*N* = 98) *n* (%) or median [IQR]	Accepted (*N* = 55) *n* (%) or median [IQR]	Non-accepted (*N* = 43) *n* (%) or median [IQR]	Accepted/Non-accepted comparison *p*-value
Age in years^a^	34 [28.5, 39.5]	34 [28, 41]	35 [29, 38]	.989
Age groups in years^b^				
18–24	8 (8.2)	7 (12.7)	1 (2.3)	.199
25–34	42 (42.9)	22 (40)	20 (46.5)	
35–44	31 (31.6)	16 (29.1)	15 (34.9)	
45–64	13 (13.3)	9 (16.4)	4 (9.3)	
55–64	4 (4.1)	1 (1.8)	3 (7)	
Gender				
Male	65 (66.3)	34 (61.8)	31 (72)	.277
Female	32 (32.6)	21 (38.2)	11 (25.6)	
Transgender^c^	1 (1)		1 (2.3)	
Education in years^a^	11 [10,12]	11 [10,12]	11 [10,12]	.646
Relationship^b^ single	81 (82.7)	43 (78.2)	38 (88.4)	.596
Disability benefits	78 (80.4)	44 (80)	35 (81.4)	.862
English speaking background	79 (80.6)	43 (78.2)	36 (83.7)	.609
Aboriginal/first nation	7 (7.1)	4 (7.3)	3 (7)	1
Ethnicity^b^				.611
European	39 (39.8)	24 (43.6)	15 (34.9)	
Oceania	33 (33.7)	17 (30.9)	16 (37.2)	

*Note*. Median and Interquartile range (IQR) were considered as data were skewed.

a Mann–Whitney *U* test.

b Fisher exact test.

c Transgender excluded from analysis.

### Clinical characteristics

Most patients in the total sample had an ICD-10 diagnosis of schizophrenia (65.3%), followed by schizoaffective disorder (32.7%). Personality traits or disorders were present in 50% of these referrals, among other comorbidities. Fifty-three percent of patients with schizophrenia had personality traits/disorders. Cluster B personality traits were most prevalent (34.3%). Neurodevelopmental conditions like Autism, Attention Deficit Hyperactivity Disorder (ADHD) or intellectual disability (ID) were present in 18.2% of the total sample (Table 2). ABI was present in 13 patients (13.2%) as a comorbid condition. Cognitive impairment was present in one-third of the total sample. Accepted and Non-accepted groups did not differ statistically regarding clinical characteristics (Table 2).

**Table 2.** Clinical characteristics of the total sample, accepted, and non-accepted groups.

Clinical diagnosis as per ICD-10	Total sample (*N* = 98) *n* (%)	Accepted (*N* = 55) *n* (%)	Non-accepted (*N* = 43) *n* (%)	Accepted/Non-accepted comparison, *p*-value
Primary diagnosis				
Schizophrenia	64 (65.3)	35 (63.6)	29 (67.4)	1.0
Schizoaffective disorder	32 (32.7)	18 (32.7)	14 (32.6)	
Bipolar disorder	1 (1)	1 (1.8)	0	
Eating disorder	1 (1)	1 (1.8)	0	
Substance use disorders	81 (82.6)	43 (78.1)	38 (88.3)	.186
Any personality traits/disorders	49 (50)	24 (45.2)	25 (58.1)	.154
ADHD^a^	9 (9.1)	8 (14.5)	1 (2.3)	
Intellectual disability^a^	7 (7.1)	3 (5.4)	4 (9.3)	
Cognitive impairment: Suspected present	32 (32.7)	21 (38.2)	11 (25.6)	.423
	26 (26.5)	13 (23.6)	13 (30.2)	
ABI (mild/mod/severe)^a^	13 (13.2)	5 (9.0)	8 (18.6)	

a Descriptive only.

### Clinical history

For the Total sample, median contact with mental health services was 14 years (IQR: 8, 20 years), median number of public hospital admissions was 12 (IQR: 7, 20.5), and the median time since first involuntary treatment was 8.5 years (IQR: 5, 15). Most referrals were received when the patient was in an acute inpatient unit (66%), with more than half having a length of stay of up to 3 months before their referral (56.1%). The median number of acute psychiatric unit admissions was 2 (IQR: 1, 5; Table 3).

**Table 3.** Clinical history for the total sample, accepted, and non-accepted groups.

Clinical variables	Total sample, *N* = 98 *n* (%), median [IQR]	Accepted (*N* = 55) *n* (%), median [IQR]	Non-accepted *n* (%), median [IQR]	Accepted/Non-accepted comparison, *p*-value
Total number of years of contact with MHS^a^	14 [8, 20]	13 [8, 20]	15 [9, 20]	.576
Time since first involuntary contact^a^	8.5 [5, 15]	8 [5, 12]	9 [5, 16]	.839
Total number of admissions prior to SECU^a^	12 [7, 20.5]	12 [7, 17]	14.5 [6, 24]	.335
Setting of patient at referral^b^				
Inpatient	66 (67.3)	45 (81.8)	21 (48.8)	Significant, χ^2^ = 16.2, *df* = 3, *p* = .001
Community	22 (22.4)	9 (16.4)	13 (30.2)	
Prison/hospital	8 (8.2)	0	8 (18.6)	
Thomas embling hospital	2 (2.0)	1 (1.8)	1 (2.3)	
Length of inpatient hospitalization before SECU^b^ referral	(*n* = 69)			.377
<1 month	31 (31.6)	19 (34.5)	12 (27.9)	
1–3 month	24 (24.5)	19 (34.5)	5 (11.6)	
3–6 month	6 (6.1)	4 (7.3)	2 (4.7)	
>6 months	8 (8.2)	4 (7.3)	4 (9.3)	
Median HoNOS^c^ scores, max score possible 48	20 [14, 23], (*n* = 64)	20 [14, 23], (*n* = 55)	24 [21, 25], (*n* = 10)	
Median LSP scores^c^, max score possible 48	22.5 [16.25, 27], (*n* = 64)	21 [15.75, 26], (*n* = 54)	28.5 [24, 35], (*n* = 10)	

a Mann–Whitney *U* test.

b Fisher exact test.

c Descriptive only.

The Non-accepted group had a median of two additional years of contact with mental health services than the accepted group. The total number of public admissions also differed by 2. Only 20% had a previous SECU referral (before 2019), and 30% had a Community Care unit admission (CCU). A higher proportion of the accepted group had a length of admission up to 3 months in an acute inpatient unit before being transferred to SECU (69%) compared to non-accepted patients. More accepted patients were inpatient (81.8% vs. 48.8%) at the time of referral compared to the Non-accepted group (Table 3).

### Substance use history

Most of the total sample had a comorbid substance use diagnosis (82%). Cannabis (77.7%) was the single most reported substance, followed by amphetamines (69.1%) and alcohol (45.6%). Multiple substance use was common, with 58% receiving the diagnosis (Median of three substances used across the lifespan).

Most of the total sample was pre-contemplative about harm minimization (67.9%), with 27% previously engaged with Drug and Alcohol services (AOD). Opioid substitution therapy (current and past) was taken up by 65% of opioid users.

Lifetime use or type of substance was not significantly different between Accepted and Non-accepted groups. The Non-accepted group had slightly more substance users (*n* = 38, 88.4%) compared to the Accepted group (*n* = 43, 78.2%). Accepted patients were twice as likely to be pre-contemplative about harm minimization and engaged with AOD services more, but this was not statistically different from the Non-accepted group.

### Trauma and child sexual abuse (CSA)

Most patients in the Total sample had a history of trauma (84.6%), and a smaller proportion had disclosed history of CSA (32.7%). Trauma experience was comparable across the Accepted and Non-accepted groups (80.4% vs. 87.8%).

### Physical health

In the total sample, most patients had a medical comorbidity (80%). A small proportion (13.3%) had more than five illnesses. Hepatic issues predominated (20.4%), followed by metabolic issues (17.3%). More than 20% of the total sample tested positive for Hepatitis C, and a small proportion tested positive for Hepatitis B (2%). The rest of the cohort was untested or had unknown hepatitis status (75%). Most referred patients from the total sample did not have a GP or did not engage with a GP (56.7%).

Controlling for age and gender, number of medical illnesses was not significantly different across Accepted and Non-accepted groups. Non-accepted patients had more Hepatic issues compared to the metabolic and cardiac risks in the accepted group. More patients had Hepatitis C in the non-accepted group. Due to the small numbers in each group, statistical analysis was not conducted. More patients from the accepted group engaged with a GP, but this was not statistically significant.

### Legal status

Most of the total sample were involuntary under the Mental Health Act (90%), with the length of orders of up to 6 months (74.5%). Around a third of patients were under guardianship orders. Ninety percent of patients did not have a nominated person or advanced statement under the Mental Health Act (MHA). A small proportion was under forensic order (Crimes Mental Impairment and Unfitness [CMIA] Act 1997 [7%]).

The involuntary legal status and length of orders were comparable between Accepted and Non-accepted groups. There were no voluntary patients in the accepted group. Most voluntary patients in the Non-accepted group were in prison given the inability for the Mental Health Act to be applied in that setting.

### Treatment history

More than 80% of the Total sample was on single depot and oral medication treatment. A small proportion (6.1%) was on double depot treatment or clozapine (7%) at the referral point. Polypharmacy was the norm rather than an exception, with 47% being on 3 to 5 psychotropic medications. One-third of all patients had discontinued clozapine by the time they were referred to the SECU. The main reason for discontinuation was nonadherence. More than two-thirds had never tried clozapine. Zuclopenthixol (oral or depot) and haloperidol (oral or depot) were preferred typical antipsychotics, and olanzapine (oral or depot) with paliperidone (oral or depot) was the preferred atypical antipsychotic. A small minority of patients (20%) had received electroconvulsive therapy in the past, with 38% of them experiencing a positive therapeutic response (Table 4).

**Table 4.** Treatment history for the total sample, the Accepted, and non-accepted groups.

Different treatment at referral	Total sample (*N* = 98), *n* (%)	Accepted (*N* = 55), *n* (%)	Non-accepted (*N* = 43), *n* (%)	Accepted/Non-accepted comparison, *p*-value
Double depot treatment^a^	6 (6.1)	1 (1.8)	5 (11.6)	.084
Single depot treatment	82 (83.7)	49 (89.1)	33 (76.7)	.101
Current clozapine	7 (7.1)	7 (12.7)	0	χ^2^ = 5.8, *df* = 1, *p* = .015
Number of psychotropic medications				
<3	46 (46.9)	25 (45.5)	21 (48.8)	.949
3–5	46 (46.9)	26 (47.3)	20 (46.5)	
>5	6 (6.1)	4 (7.3)	2 (4.7)	
Clozapine discontinued^a^	29 (29.6)	12 (21.8)	17 (39.5)	χ^2^ = 7.3, *df* = 2, *p* = .25
Never been clozapine	65 (66.3)	42 (64.6)	23 (35.4)	χ^2^ = 5.6, *df* = 2, *p* = .59
ECT	21 (21.4)	11 (20)	10 (23.3)	.805
Response to ECT-good^b^ (*n* = 21)	8 (38)	6 (60)	2 (20)	

a Fisher Exact test.

b Descriptive.

None of the Non-accepted patients were on clozapine at the point of referral, and they were more likely to be on double-depot treatment. The preferred typical and atypical antipsychotics (first and second) did not differ between Accepted and Non-accepted groups.

### Risk profile

Sixty percent of patients in the total sample had a combination of risk to self and others, followed by harm to others (31.6%) and risk to self (7.1%). Among risk to self, self-neglect and sexual vulnerability ranked highest, followed by severe self-harm and suicidal attempts. In terms of harm to others, physical threats and violence with sexual harassment and violence ranked high; perpetration of family violence and weapons-related violence ranked next.

Controlling for age group and gender, there were no significant differences in risk profile across Accepted and Non-accepted patients.

## Discussion

The current study is the first to describe the characteristics of patients referred over a 5-year period to a SECU in Australia. Among the 98 first-time referrals studied, 56% were accepted into the program.

The SECU study findings were generally consistent with previous related studies from the United Kingdom and Australia (Huang et al., 2023; Killaspy, Marston, et al., 2016; Lavelle, 2007; Melzer et al., 2004a). Socio-demographic factors like gender, ethnicity, education, relationship, parenthood, family support, and financial status matched the studies above. Clinical characteristics, including diagnosis of Schizophrenia, physical health and choices of substances overall matched the profile of patients in those studies.

Most of the patient group was between 25 and 34 years old. This is a younger cohort compared to the waitlisted patients in the Irish rehabilitation study (Lavelle, 2007), community patients (Davies et al., 2023; Harvey et al., 2012) and rehabilitation users (Killaspy, Marston, et al., 2016). The proportion of First Nations, Culturally and Linguistically Diverse (CALD) and Lesbian Gay Bisexual Trans Intersex and Queer (LGBTIQ) was a minority in the total sample, which could be related to the low population percentage in the serviced area of the Austin SECU and under reporting of LGBTIQ, in-line with findings of Davies et al. (2023).

Diagnostic comorbidity was high. This study found a higher proportion of personality, neurodevelopmental comorbidity and cognitive impairment compared to Huang et al. (2023) and Killaspy, Marston, et al. (2016). It does replicate the finding that personality disorder exists as a comorbidity with schizophrenia (Moore et al., 2012; Wei et al., 2016). Personality disorder comorbidity is usually higher in medium secure units (30%) and high secure units (45%; Huang et al., 2023). Hence, this was a surprising finding, given that the study involved a SECU unit, which is low-secure.

PTSD was not a significant diagnosis in the total sample for the SECU study, despite the finding that trauma experience was high. This is in keeping with the study by Harvey et al. (2016). There are several factors reported for under-detection and underdiagnosis of PTSD (Lommen & Restifo, 2009) This includes PTSD not being the presenting complaint, and patients and clinicians being reluctant to revisit trauma or not recognizing the relevance of any prior trauma to the current presentation. This is further compounded by poor documentation of assessments and findings. Our study provides evidence that trauma must be recognized as one of the contributing factors toward complexity in psychosis, just as unremitting illness and functional decline, comorbidity, substance use, physical illness, and cognitive impairment (Lommen & Restifo, 2009).

Physical comorbidity was present in the total sample, with 21% having Hepatic illness. This is based on a risk-based approach to screening (like intravenous drug use and sexual exposure). This rate is much higher than the 6% reported by Davies et al. (2023) and is higher than the general population (Braude et al., 2021). Reasons could include substance use, social disadvantage, cognitive and positive symptoms related to SMI, stigma, misconceptions of healthcare workers and diagnostic overshadowing (Braude et al., 2021; Preston et al., 2024). This is in keeping with the socio-economic and clinical status of the SECU study sample. SECU follows a risk-based approach to detection. Australia-wide, this approach has failed to significantly increase diagnosis and treatment conversion rates over the past decade (Allard et al., 2021; Braude et al., 2021). Currently, inpatient Hepatitis C treatment is not funded by the Australian Pharmaceutical Benefits Scheme. Access to treatment is through community linkage (Braude et al., 2023). Micro-elimination strategies have been successful in settings like Prisons and vulnerable cohorts, but mental health has lagged behind (Braude et al., 2023; Gofton et al., 2024).

Chronicity of mental illness was understandable; a median contact of 14 years with mental health services, a median of 8.5 years since the first involuntary mental health treatment and a median number of 12 hospitalizations after first contact with mental health services. All patients had at least one involuntary treatment in the past. This is a much higher hospitalization rate compared to findings by Killaspy, Marston, et al. (2016) and Lavelle (2007). CCUs are underutilized in this sample. This indicates that the offer of SECU referral happens later in mental health management trajectory.

Self-neglect, sexual vulnerability, and severe self-harm ranked high among risks to self in the total sample. Physical threats and violence, with sexual harassment and family violence, ranked high in risk to others. These findings are similar to Killaspy, Marston, et al. (2016). Moore et al. (2012) found that comorbidity between personality and schizophrenia leads to worse clinical outcomes in terms of increased suicidality, poor cognition and experience of childhood trauma. The high comorbidity with cluster B personality traits may explain high self-harm, sexual vulnerability, and violence risks in this SECU total sample.

The SECU setting provides care to both perpetrators of family violence and serious assaults, and to patients who are victim survivors of sexual or FV prior to referral. This creates challenges in managing exposure and containment of risks within a restrictive and multi-gendered setting and raises ethical concerns due to the limitation of freedom and autonomy. The inpatient and involuntary setting of treatment brings into play an imbalance of power and control, managing risk whilst providing therapeutic environments. Such an environment may cause iatrogenic trauma (M. L. Edwards & Morris, 2024). Working with trauma in such a setting requires enhancing practitioners’ knowledge and skills in building trust with vulnerable patients, balancing restrictive treatment of mental health conditions and risk management whilst providing an environment of safety, compassion and validation (M. L. Edwards & Morris, 2024). Recent Safewards initiatives in the SECU environment positively impacted both patient and staff experiences and is a step towards making these environments safe and respectful and increasing patient participation in their care with dignity and hope (Fletcher et al., 2019).

Polypharmacy in treatment was the norm rather than the exception in the current study. Morgan et al.’s (2017) findings of the SHIP study were that two-thirds were on more than one medication. Davies et al. (2023) reported 37% had polypharmacy in their study. A small proportion of patients in the total sample received double-depot treatments, which are off-label treatments and may reflect both desperation and convenience in psychopharmacology treatment. Double-depot treatment tends to improve compliance with medication but requires close physical health monitoring of extra side effects (Lenardon et al., 2017).

Surprisingly, two-thirds of the total sample never tried clozapine, and only 20% received ECT, which is similar to Morgan et al. (2017) and Davies et al. (2023). One of the reasons for referral to SECU is to start patients on clozapine. The SECU study didn’t examine factors that could have contributed to this. Farooq et al. (2019) systematic review into barriers to using clozapine indicated several factors such as age > 20, polypharmacy, clinicians’ perception of likely non-adherence in patients, shortage of beds, and service fragmentation. These factors were observed in the SECU study, which then supports the observation of underutilization and delayed initiation of clozapine in the literature (Chakrabarti, 2021). Unfortunately, this leads to the postponement of initiation until admission to SECU. Delayed initiation has been associated with a higher risk of re-hospitalization, poor outcomes, and longer stays in inpatient units (Hatano et al., 2023; Shah et al., 2018).

Patients with schizophrenia make up only a small proportion of patients receiving ECT in Australia (Das et al., 2019; Leiknes et al., 2012). Changes in the Mental Health Act (MHA 1986–MHA 2014) have seen a reduction in patients with psychosis and ECT given under involuntary consent (Das et al., 2019). Chen et al. (2024) observed that declining hospital mental health beds and the cost of inpatient psychiatric care have been disincentives to the provision of ECT to publicly funded patients, in contrast to private patients. Being unmarried and living in socioeconomic disadvantage independently reduced the odds of receiving ECT treatment in their study (Chen et al., 2024). The SECU patient cohort belongs to such a group. Birong et al. (2023) suggested ECT may be considered early in the course of illness, which then has a high chance of an effective response in patients with schizophrenia. Augmentation with ECT has been noted to have a faster response among patients with a high risk of aggression and self-harm (Chakrabarti, 2021). This appears to be underutilized in the total sample.

### Implications for practice and future research

SECU serves as a single point of long-term inpatient rehabilitation for all non-forensic patients within Victorian public mental health services. Therefore, the expectation of high comorbidity, substance use, and risk history is the norm. The absence of medium secure units in Victoria means patients who cannot be managed in the community or cannot find a bed in a high secure unit and do not meet criteria under the Secure Treatment order or the Crimes Act, are being referred to SECU. The creation of medium and high-secure civil units or subsections within current SECUs must be considered. Increasing the number of low-secure beds within Forensic services is another consideration.

Integration of CCU with SECU would help to ensure that rehabilitation gains made during SECU stay are not lost immediately post-discharge. The co-location of future SECUs and CCUs needs to be considered so that step-ups and step-downs can become seamless between these programs. A current requirement is for patients to step up to SECU through inpatient units, which blocks an acute inpatient bed and creates delays in the transfer of patients, as can be seen in up to 3 months of stay in an acute inpatient unit in our study. A model that flexibly uses CCU and SECU beds for long-stay patients from acute inpatient units with rapid transfer to SECU should be considered.

The diagnosis of Hepatitis B and C in this population is an important finding. WHO has set ambitious plans to eliminate hepatitis C by 2030 (Braude et al., 2023). To achieve this target, public mental health will need to move from its current risk-based approach to universal screening and offer treatment.

The low utilization of clozapine and ECT in the total sample is an important observation. Educational strategies should be implemented to address the perception of clozapine as a high-risk medication, improve access to assertive treating teams to strengthen medication adherence in the community, and explore the potential role of point-of-care testing as a more acceptable alternative to traditional blood monitoring methods (Farooq et al., 2019). A targeted approach with the assistance of people with lived experience to improve knowledge and attitude toward ECT in medical and nursing students has shown benefit (Sarma et al., 2023). Co-designing educational materials and wider circulation using social media is important to managing stigma and concerns among practitioners, families, and patients.

Further studies should examine the barriers to clozapine and ECT prescription through qualitative studies in this population to better understand their utility.

Future qualitative research could explore in-depth the reasons why patients were not accepted. This might involve interviewing or running focus groups with SECU staff and providing vignettes to explore decision-making. To complement these data, observations could be made of meetings when decisions about referrals are made.

Future studies should focus on patients’ attitudes toward inpatient rehabilitation, readiness for rehabilitation, and the determinants of successful rehabilitation in an involuntary hospital setting.

Future models of care for this cohort would need to be a one-stop shop of integrated rehabilitation. For this to be realized, SECUs should consider an adequate staffing skill mix with the addition of neuropsychologists and behavioral psychologists to help develop a person-centered plan that allows patients to exercise choice and control and learn independent living skills. Focus should be placed on therapy that considers attachment style, cognitive compensatory and remediation strategies, and family intervention. The complexity of the patient group means the treatment plans must be interdisciplinary to create a holistic understanding of complex issues.

### Limitations

Data were obtained from only a single service (Austin SECU). Austin SECU covers a significant catchment area in metropolitan Melbourne. However, the ability to generalize these results to other low-secure hospital samples is limited. The study could not compare complexity to a cohort of community patients not referred to the service to ascertain the difference. Further, follow-up of non-accepted patients’ post-referral could have provided greater insights into the unmet needs of this patient population.

The retrospective nature of the research has limitations, such as bias and missing data for certain variables. Data were missing for some of the variables for the non-accepted group because, in many cases, patients dropped out of follow-up of the service, so CMI information could not be accessed, or the service could not provide the information within the data collection period of the study. This is likely to bias the results, particularly findings for the non-accepted group. Attempts were made to collect missing data by contacting individual services with variable success. Reasons for the decline of acceptance were not available for most of the non-accepted group, which could have helped understand decision-making better.

CMI data can be unreliable. Clinicians sometimes record the main clinical presentation cross-sectionally rather than as a longitudinal and comprehensive analysis of all the multiple diagnoses. The recording of diagnoses on the CMI database can be added by clinicians of any discipline, not just those with psychiatric or psychological qualifications. This may account for discrepancies between recording trauma and substance use and receipt of formal diagnoses of PTSD or Substance Use Disorders if recording clinicians are not suitably qualified to diagnose.

Nomination of patients by the referring services may inadvertently bias acceptance data. A referring agency is unlikely to recommend a patient who does not meet SECU’s criteria, is too acutely unwell, or does not present with complex and enduring treatment needs. Conversely, given the secure, locked nature of the unit, services are more likely to refer patients who cannot be safely managed in the community and require a restricted environment. This may have resulted in inflation in non-accepted referrals, particularly those received from forensic or prison services on the grounds that the individual is determined to be too high a risk for the low secure unit to manage.

The study did not characterize risk into an ordinal scale (mild, moderate, severe) which could have helped predict acceptance into the program. However, it was reassuring to see that risk characteristics did not differ between accepted and non-accepted patients.

The re-referrals group had a small sample size. Therefore, a statistical comparison with this subgroup was not done.

## Conclusions

This study provides valuable information about patients referred to a SECU program. The patients referred had significant comorbidity with personality disorder, neurodevelopmental, physical health and substance use. Most had repeated hospitalization, and a subset had been incarcerated.

Austin SECU is meeting its mandate of accepting high-risk patients who have failed community management. The study makes the case for creating medium and high secure units within public non-forensic mental health services and increased accessibility of low-secure beds within the forensic system.

Findings may guide the clinical governance model of SECUs and potential directions for integrating CCUs and SECUs to allow a seamless transition between these programs.

The study makes a case for Universal screening of patients for Bloodborne Hepatitis and treatment for this cohort in an inpatient setting. SECU referral is appropriate for consideration of clozapine and ECT initiation and maintenance in this cohort.

The study highlights the need for a new model of care that integrates disciplines, creates holistic treatment plans, and acts as a one-stop shop for rehabilitation to address patients’ complex physical, psychological, and social needs.

## Supplemental Material

sj-docx-1-isp-10.1177_00207640251343171 – Supplemental material for Social demographics and clinical characteristics of referred adult mental health patients to an Australian secure extended care unit: A 5-year retrospective study Supplemental material, sj-docx-1-isp-10.1177_00207640251343171 for Social demographics and clinical characteristics of referred adult mental health patients to an Australian secure extended care unit: A 5-year retrospective study by Partha Das, Emma Robertson, Victoria Harpwood and Stephanie Tierney in International Journal of Social Psychiatry
