# Supplementary material for: Social demographics and clinical characteristics of referred adult mental health patients to an Australian secure extended care unit: A 5-year retrospective study
Source: Int J Soc Psychiatry. 2025 Jun 12;71(8):1489–500. doi: 10.1177/00207640251343171 (PMC12634904; doi:10.1177/00207640251343171)
Supplement: sj-docx-1-isp-10.1177_00207640251343171 – Supplemental material for Social demographics and clinical characteristics of referred adult mental health patients to an Australian secure extended care unit: A 5-year retrospective study [file sj-docx-1-isp-10.1177_00207640251343171.docx]

Supplementary tables

SI: Clinical history for the Total sample, Accepted and Non-accepted groups.

| Clinical variables | Total Sample  N=98  n (%)  Median [IQR] | Accepted (N=55)  n (%)  Median [IQR] | Non-accepted  n (%)  Median [IQR] | Accepted/Non-accepted comparison  p-value |
| --- | --- | --- | --- | --- |
| Median admission to inpatient in last 12 months | 2[1,5] | 2.5[1,5] | 2[1,4] | 0.254 |
| Previous SECU referral | 26(26.5) | 15(28.3) | 11(25) | 0.851 |
| Previous SECU admission | 20(20.4) | 11(20.0) | 9(20.9) | 0.910 |
| Previous CCU referral | 30(30.6) | 19(34.5) | 11(25.6) | 0.339 |
